# Intercropping Maize–Faba Bean Reduced Yield and Economic Losses Caused by *Busseola fusca* (Fuller) in Semiarid Area

**DOI:** 10.1155/2024/4929479

**Published:** 2024-10-29

**Authors:** Tesfay Gidey, Daniel Hagos Berhe, Emiru Birhane, Haftu Abrha, Yirga Gufi

**Affiliations:** ^1^Department of Plant Science, College of Agriculture and Environmental Sciences, Adigrat University, P.O. Box 50, Adigrat, Ethiopia; ^2^Department of Natural Resources Management, College of Agriculture and Environmental Sciences, Adigrat University, P.O. Box 50, Adigrat, Ethiopia; ^3^Department of Land Resource Management and Environmental Protection, College of Dryland Agriculture and Natural Resources, Mekelle University, P.O. Box 231, Mekelle, Ethiopia; ^4^Institute of Climate and Society, Mekelle University, P.O. Box 231, Mekelle, Ethiopia; ^5^Faculty of Environmental Sciences and Natural Resource Management, Norwegian University of Life Sciences (NMBU), As, Norway; ^6^PASET Regional Scholarship and Innovation Fund (RSIF), International Centre of Insect Physiology and Ecology (ICIPE), P.O. Box 30, Nairobi 772-00100, Kenya; ^7^Tigray Institute of Policy Studies, P.O. Box 902, Mekelle, Ethiopia

**Keywords:** Ethiopia, grain yield, intercropping, plant density, stemborer, yield loss

## Abstract

Intercropping is an important practice for controlling crop pests in Ethiopia. However, there is a limited studies on the maize–faba bean intercropping benefits for controlling stemborer (*Busseola fusca*) pests. This study was carried out at Wukro Agricultural College, Tigray, Ethiopia, to investigate the effects of maize intercrops with two faba bean varieties (Gora and Moti) at three different population levels (25%, 50%, and 75%) of the recommended sole faba bean (250,000 plants ha^−1^) on infestation, density, and damage of stemborer on maize. The intercrops significantly (*p* < 0.05) reduced the stemborer infestation, density, and damage on maize over to the sole maize. Maize intercrops with the Gora faba bean variety at a density of 50% lowered the pest damage on maize cobs by 35% relative to the sole maize. The intercrop also reduced the grain yield and economic losses due to the pest by 48% and 53%, respectively, compared to the sole maize. The results recommended that maize intercropped with the Gora faba bean variety at a density of 50% could be used as an alternative farming against stemborer pest in the semiarid farming systems of northern Ethiopia.

## 1. Introduction

Maize (*Zea may* L.) is one of the most important staple food crops in Africa. It is an important food source for over 300 million Africans [1]. In 2017, 40 million ha of African farms were covered by maize, representing 24% of the total cultivated areas. From this, over 75 million tons were harvested [1]. Globally, between 1981 and 2008, maize total production increased on average by 1.8% annually [2]. However, its grain yields in some East African countries have decreased over the last 2 decades [3] because of both biotic and abiotic pressures [4].

The lepidopteran stemborers such as *Chilo partellus* (Swinhoe) and *Busseola fusca* (Fuller) are the main biotic factors affecting maize production in Africa [5–7]. These pests are causing a substantial maize gain yield losses across Africa, for instance, 14% and 57%, respectively, in Kenya and Ethiopia [6, 8, 9]. In connection with, e.g., in Kenya, the economic loss *of* the pest was estimated to be US$76 per year [10]. In the past 2 decades, studies focused on the biological and habitat management strategies for stemborer control. The habitat management strategies included soil nutrients management [11], crop rotation [12], trap plants [13], push–pull technology [14, 15], and intercropping [6, 12, 15–18].

Intercropping is commonly practiced in African subsistence farming systems to improve crop yield, soil fertility, and economic revenue [19–22]. It also reduces risks of insects and pests damage on crops [14, 23, 24]. In the African smallholder cereal production systems, intercropping is reported to decrease stemborer damage by 83% over monoculture maize [5, 8, 16] by reducing the host for ovipositing of the pest and increasing the abundance of natural enemies [15, 16, 25]. Besides, the benefits of intercropping to control pests can be enhanced by improving crop management strategies such as planting proportion, time and arrangements, and cultivar selection [5–7, 17, 26]. The use of optimum plant density of a nonhost legume crop with maize, e.g., would create an obstacle for the movement of stemborer, thereby reducing its host finding success and oviposition preference [7, 17, 27, 28]. The effectiveness of maize intercrops with a legume crop to control the pest also depends on their compatibility with the use of growth resources such as light, water, and nutrients [27, 29–31].

In Ethiopia, maize was ranked as the second crop in terms of area coverage, representing 24% of the total areas given for cereal production [32]. It is cultivated by 9 million farmers under various agroecological and socioeconomic situations [33]. Continuous cultivation of improved maize varieties in Ethiopia over the last 30 years changed farming systems from heterogeneity to maize homogeneity. This monoculture practice increasingly created suitable environmental conditions for stemborer infestation and damage that resulted in grain yield losses of up to 100% [6, 8, 34]. Despite this, because of the increasing cost of insecticides and their adverse effects on nontarget beneficial insects (e.g., honey bee) and human health [15], the farmers have looked at alternative interventions such as inclusion of leguminous crops (e.g., faba bean) into maize-based farming systems to enhance crop grain yield [35]. However, farmers frequently utilize excessive plant population, and faba bean varieties that are not well-suited for maize-based farming systems [7]. Therefore, this study was carried out to examine the effects of intercropping maize with different faba bean varieties at three population levels on 1) stemborer infestation and population density and 2) stemborer-related damage on maize grain yield and overall economic returns.

## 2. Materials and Methods

### 2.1. Description of the Study Area

In the main cropping season of 2019, we carried out a field experiment for the study at Wukro Agricultural College, Eastern zone of Tigray Region, Ethiopia. The area is found within the vicinity of Wukro town at 13°43′ N latitude and 39°25′ E longitude and is distanced 40 km south ward of Mekelle city, the capital of Tigray. It is characterized by semiarid climates. Within the study area, the elevation ranges between 1900 and 2000 m.a.s.l with a monthly average minimum temperature of 10°C and average maximum temperature of 32°C. Its annual rainfall varying from 350 to 850 mm and mainly concentrates between June and September. The area commonly cultivates crops such as maize, wheat, barley, faba bean, and chickpea. The major crop pests and diseases in the area include stemborer, wheat rust, and chocolate spot with different weed species [22]. The textural soil class of the area is sandy loam with proportions of 9% clay, 12% silt, and 79% sand. The pH of the soil is 8.2. The soil comprised 0.49 (%) organic carbon, 0.43 (%) total nitrogen, and 29.1 (%) available phosphors [22]. The experimental field was ploughed twice using a tractor to prepare a good seedbed for the study.

### 2.2. Experimental Design and Field Management

The study consisted of seven treatments: the recommended population of maize (44,444 plants ha⁻^1^) intercropped with two faba bean varieties (Gora and Moti) at three population levels (25%, 50%, and 75% of the recommended population of 250,000 plants ha⁻^1^). Sole maize was also included as control. Both Gora and Moti are early maturing varieties, needed approximately 140 days to reach physiological maturity. However, they are different in height: Gora is shorter while Moti is medium in height. For maize, the improved BH-543 variety, an early maturing with medium plant height, was used. We got seeds of the crops from Tigray Agricultural Research Institute.

Maize in the intercrops and sole used a plot size of 3.75 m × 5.1 m. The intercrop plots had five rows of maize; within them, four rows of faba bean were intercropped. The sole maize plots comprised only five rows of maize plants. The recommended spacing of 75 cm between rows and 30 cm between plants was used for maize. Spacing for the faba bean rows was adjusted according to the planting density, 39 cm for a density of 25%, 20 cm for a 50%, and 13 cm for a 75%. Three central rows of maize and two rows of faba beans with a net plot area of 7.65 m^2^ were used for data collection [35]. Sole faba bean used a plot size of 2.4 m × 5.1 m, comprising six rows of faba beans spacing 40 cm between the rows and 10 cm between plants. Data collection focused on the two central rows, covering a net plot area of 2.04 m^2^ [36]. A randomized complete block design with three replications was used. Plots of the intercrops and sole maize received the recommended fertilizer rates of 41/20 kg NP ha⁻^1^ at the planting stage and 23 kg N ha⁻^1^ at the knee-heightstage of maize. Sole faba bean plots were only fertilized with 18/20 kg NP ha⁻^1^ at the planting stage. All the necessary agronomic practices were applied uniformly to the plots following the local guidelines. Both the crops were manually planted simultaneously on June 13, 2019, and separately harvested on October 18, 2019.

### 2.3. Data Collection and Analysis

The stemborer damage on maize was determined using nondestructive and destructive sampling techniques. From each net plot area, the percent infestation and leaf feeding score of the pest were recorded twice during the vegetative stages of maize, at 20 and 60 days after emergence (DAE) of the maize. A scale of one to nine scale developed by Sharma et al. [37] was followed to score the leaf feeding levels, and 1 represents healthy plants, while nine is heavily damaged. For scoring, the shot holes or windows on the surface of leaves of individual maize plants caused by the pest were considered [37]. Percent infestation was calculated by comparing the infested plants to the whole plants within the net plot [17]. Using this procedure, at the harvest stage of the maize crop, the percent infestation was again calculated. Moreover, five plants from each net plot were randomly sampled and dissected to record plant height, and the pest damages include percent tunnel length, holes per plant, borer numbers per plant, and percent cob damage. Tunnel length on maize stalk caused by the pest was measured using a ruler and converted into percentages, comparing with the whole plant height [6]. The pest larvae were reared and fed fresh stem pieces of maize every 4 days in the plastic Petri dishes at the laboratory until adult stemborer emergence to determine borer numbers [6, 17]. Percent cob damage was calculated by comparing the surface area damaged by the pest to the total surface area of the cob [38].

Furthermore, in each net plot area, we harvested all maize and faba bean plants, and then, the grains (nondamaged and damaged) were adjusted to 12.5% moisture content for maize and 10% for faba bean [17] to calculate grain yield losses caused by the pest. To compute economic losses of the pest, the gross monetary values (GMV) for each treatment were calculated based on the average prices of the grain yields of the crops at local market (maize was priced 35 USD per quintal, and faba bean about 80 USD). For analysis, the grain yields and economic losses were converted into hectare basis.

Data were analyzed using analysis of variance (ANOVA). Least significant difference (LSD) at the 5% probability level was performed to establish difference among the treatment means. Only the interaction effects of the treatments were reported in the study as the effects of the main factors were not significant. The statistical analyses were performed using the statistical analysis software (SAS) Version 9.2.

## 3. Results

### 3.1. Stemborer Infestation and Density

Effects of cropping systems on percent infestation at the vegetative stage, leaf feeding score, percent infestation at harvest, percent tunnel length, and bores per plant were significant (*p* < 0.05). However, they did not significantly affect plant height and holes per plant (Tables 1 and 2). Percent infestation and leaf feeding scores at 20 and 60 DAE were higher in the monoculture maize over in the intercrops (Table 1). The sole maize had a higher infestation level, percent tunnel length, and borers per plant than the intercrops (Table 2). Compared to the sole maize, maize intercropped with the Gora variety at a density of 50% decreased the infestation level of the pest at the vegetative (20 DAE) and harvest stages by 37% and 25%, respectively (Tables 1 and 2). It also reduced stemborer density by 39% over the sole maize (Table 2).

### 3.2. Stemborer Damage

Effects of the cropping systems on percent cob damage, grain yield, and economic losses were significant (*p* < 0.05) (Table 3). The stemborer damage on maize cobs was 4.4%–6.8%. It also caused grain yield losses between 10.1% and 19.6%, and its associated economic losses were about $134–288 ha^−1^ (Table 3). These damages were higher on the sole maize than on the intercrops (Table 3), and these would be associated with the presence of high infestation levels, tunnel length, and stem bore density in the sole maize (Tables 1 and 2). Maize when intercropped with the Gora variety at a density of 50% lowered the pest damage on maize cobs by 35% and reduced the grain yield and economic losses by up to 48% and 53%, respectively, over the sole maize (Table 3).

## 4. Discussion

The study showed that the intercrops significantly reduced stemborer damage on maize compared to the sole maize. For example, the intercrops lowered stemborer density from 28% to 38% over the sole maize. This would be the main reason for reducing maize grain yields (by 48%) and economic (by 53%) losses in the intercrops over the sole maize. This is in agreement with Wale et al. [6] who reported a reduction of stemborer density by 22%–63% under maize intercrops with different legume crops relative to monoculture maize in northern Ethiopia. In connection with this, maize yield loss in the intercrops was estimated to be 22% lower than the monoculture maize [6]. Maize intercrops with haricot beans provided 82% lower borer density than the sole maize in central Ethiopia [17]. Similarly, 20% lower borer density was found under maize and cowpea intercropping relative to the sole maize in Kenya. Consequently, this raised the total maize grain yield by 42% in the intercrops over the sole maize [39]. In Kenya, 55% lower borer density was found under maze and haricot intercropping compared to the monoculture maize. Due to this reason, the intercrops provided higher grain yields while lowering economic losses over the sole maize [40]. Furthermore, maize intercrops with desmodium grass decreased maize yield loss by 75% relative to the sole maize [41].

Intercropped maize with the Gora variety at a density of 50% provided the highest benefits in controlling stemborer pests (expressed in terms of lower grain yield and economic losses) compared to the sole maize. This may be explained by a better compatibility of the crops at this density when focusing on the growth resources such as water and nutrients due to the good morphological characteristics of the Gora variety (e.g., early maturity). This agrees with Belay et al. [17] who found that maize intercrops with haricot bean at a density of 50% raised total grain yields and economic returns while reducing the pest damage on maize relative to the sole maize. Similarly, the inclusion of lablab at optimum density (8 plants m^−2^) into maize-based cropping systems decreased the pest infestation and density [28]. Besides, Ndemah et al. [25] reported the reverse relationship between the nonhost plant density and the density of the pest was probably due to challenges causing for the female moth to find host crops for oviposition.

The study found a lower stemborer infestation and density in the intercrops over the sole maize. In line with this, a lower grain yield and economic losses were recorded in the intercrops. This could be due to the presence of the nonhost faba bean with maize may reduce the host finding of the ovipositing stemborer females. It could also decrease the chances of the dispersing larvae of the pest landing on a suitable host, thereby increasing larval mortality. Further, the intercrops could increase the abundance of natural enemies (e.g., ants) against the stemborer. Due to these justifications, the role of intercropping as a strategy for controlling stemborer in maize-based farming systems has been reported elsewhere [6, 11, 12, 16, 18, 25, 30, 31]. However, maize and common bean intercrops did not significantly reduce stemborer infestation at the landscape level due to the low population of common bean, which was too low to affect the host crop finding of stemborer females for oviposition [7].

## 5. Conclusions

The study indicated that intercropping maize with two faba bean varieties (Gora and Moti) at three population levels (25%, 50%, and 75%) of the recommended sole faba bean significantly lowered stemborer infestation, density, and damage on maize grain yield compared to the sole maize. Intercropping maize with the Gora faba bean variety at a density of 50% lowered the pest damage on maize grain yield and economic losses by 48% and 53%, respectively, over the sole maize. Thus, this intercropping combination could be scaled up as an alternative farming strategy against stemborer pests for maize productivity in the semiarid areas of northern Ethiopia. However, additional studies should be conducted in different areas in the semiarid of Ethiopia to substantiate the results of this research.

## Figures and Tables

**Table 1:** Effects of intercropping maize with faba bean varieties at three population levels on stemborer infestation and leaf feeding.

Treatments	Infestation (%)		Leaf feeding scores^1^	
	20 DAE	60 DAE	20 DAE	60 DAE
Sole maize	10.6^a^	15.6^a^	4.3^a^	5.2^a^
Maize with GFB at 25%	8.2^d^	10.2^ef^	3.3^bcd^	4.1^bc^
Maize with GFB at 50%	6.8^e^	9.8^f^	3.1^cd^	3.8^c^
Maize with GFB at 75%	9.1^c^	10.9^de^	3.1^cd^	4.3^b^
Maize with MFB at 25%	8.9^c^	11.4 ^d^	3.5^bcd^	4.8^a^
Maize with MFB at 50%	8.3^d^	12.6^c^	3.8^ab^	4.9^a^
Maize with MFB at 75%	10.1^b^	13.9^b^	3.6^bc^	4.1^bc^
SEM±	0.15	0.29	0.18	0.16
LSD (5%)	0.46	0.89	0.54	0.46
CV (%)	3.0	4.2	8.9	6.1

*Note:* Means within the column used the same letter(s) are not significantly different at a 5% probability level.

Abbreviations: CV = coefficient of variation, DAE = days after emergence of maize, GFB = gora faba bean variety, LSD = least significant difference, MFB = moti faba bean variety, SEM = standard error for means.

^1^On a scale of 1–9 [37].

**Table 2:** Effects of intercropping maize with faba bean varieties at three population levels on maize plant height and different stemborer damage parameters.

Treatments	Plant height (cm)	Infestation (%)	Tunneling (%)	Holes plant^−1^	Borers plant^−1^
Sole maize	153	19.9^a^	2.3^a^	1.2	1.8^a^
Maize with GFB at 25%	156	15.8^cd^	1.8^b^	1.1	1.1^bc^
Maize with GFB at 50%	158	15.0^d^	1.6^b^	1.2	1.1^c^
Maize with GFB at 75%	156	17.2^bc^	1.9^b^	1.2	1.1^bc^
Maize with MFB at 25%	152	18.1^ab^	1.8^b^	1.1	1.3^b^
Maize with MFB at 50%	151	17.5^bc^	1.8^b^	1.2	1.2^b^
Maize with MFB at 75%	150	16.7^bc^	1.7^b^	1.3	1.1^bc^
SEM±	2.89	0.63	0.12	0.11	0.09
LSD (5%)	NS	1.91	0.37	NS	0.26
CV (%)	3.3	6.3	11.6	16.6	12.5

*Note:* Means within the column used the same letter(s) are not significantly different at a 5% probability level.

Abbreviations: CV = coefficient of variation, GFB = gora faba bean variety, LSD = least significant difference, MFB = moti faba bean variety, NS = nonsignificant, SEM = standard error for means.

**Table 3:** Effects of intercropping maize with faba bean varieties at three population levels on stemborer damage on maize cobs, grain yield, and economic revenues.

Treatments	Cob damage (%)	Maize grain yield (quintal ha^−1^)	Yield loss (%)	Economic loss (USD ha^−1^)
Sole maize	6.8^a^	42^a^	19.6^a^	288.4^a^
Maize with GFB at 25%	4.4^b^	40.5^a^	11.5^c^	163.0^bc^
Maize with GFB at 50%	4.4^b^	38^ab^	10.1^d^	134.3^e^
Maize with GFB at 75%	4.7^b^	28^c^	13.8^c^	135.2^e^
Maize with MFB at 25%	5.1^b^	33.7^bc^	13.9^b^	164.0^b^
Maize with MFB at 50%	4.9^b^	32.4^bc^	13.6^b^	154.2^cd^
Maize with MFB at 75%	4.7^b^	31.5^bc^	13.3^b^	146.6^d^
SEM±	0.36	2.30	0.31	2.94
LSD (5%)	1.10	6.90	0.93	8.93
CV (%)	12.6	13.0	3.9	3.0

*Note:* Means within the column used the same letter(s) are not significantly different at a 5% probability level.

Abbreviations: CV = coefficient of variation, GFB = gora faba bean variety, LSD = least significant difference, MFB = moti faba bean variety, SEM = standard error for means.

## Data Availability

The data used to support our findings of this study are available from the corresponding author upon reasonable request.
